# Proteomic dataset: Profiling of glioma C6 and astrocytes rat cell lines before and after co-cultivation

**DOI:** 10.1016/j.dib.2021.107658

**Published:** 2021-12-01

**Authors:** Artemiy S. Silantyev, Olga N. Bukato, Ivan O. Butenko, Anastasia A. Chernysheva, Olga V. Pobeguts, Alexander E. Nosyrev, Olga I. Gurina

**Affiliations:** a Federal State Institution V. P. Serbsky Federal Medical Research Center of Psychiatry and Narcology National Scientific Research Centre on Addictions of the Ministry of Healthcare of the Russian Federation, Moscow 119002, Russian Federation; b Federal Research and Clinical Center of Physical-Chemical Medicine of Federal Medical Biological Agency, Moscow 119435, Russian Federation; c Center of Bioanalytical Research and Molecular Designm, FSAEI HE I.M. Sechenov First MSMU MOH Russia, Sechenov University, Moscow 119048, Russian Federation; d Department of Fundamental and Applied Neurobiology, Serbsky National Research Center for Social and Forensic Psychiatry, Ministry of Health and Social Development of the Russian Federation, Moscow 119034, Russian Federation

**Keywords:** Glioma C6, Co-culture, LC-MS/MS, Proteome

## Abstract

Human multiforme glioblastoma is characterized by an unfavorable prognosis, low survival rate and extremely limited possibilities for therapy. Rat C6 glioma is an experimental model for the study of glioblastoma growth and invasion. It has been shown that the growth and development of the tumor is accompanied by changes in the surrounding normotypic tissues [1]. These changes create a favorable environment for the development of the tumor and give it an evolutionary advantage [2]. Description of changes occurring in normotypic cells of the body upon their contact with tumor cells is of great interest. We have grown C6 glioma cells and rat astrocytes, as well as astrocyte cells co-cultured together with C6 glioma. We performed proteome-wide LC-MS analysis of these experimental groups. The data includes LC-MS/MS raw files and exported MaxQuant and ProteinPilot search results with fasta. Dataset published in the PRIDE repository project accession PXD026776.

## Specifications Table

**Table:** 

Subject	Biology
Specific subject area	Proteomics
Type of data	LC-MS/MS data and identification data
How data were acquired	Microflow HPLC system Eksigent Ekspert (Sciex, USA) with autosampler Eksigent Ekspert 400 (Sciex, USA) coupled to ABSciex 6600 with a microflow AB Sciex Duo Spray Ion Source (Sciex, USA)
Data format	Raw and analyzed data
Parameters for data collection	We analyzed glioma C6, rat astrocytes co-cultured with glioma C6, rat astrocytes (control).
Description of data collection	Dataset covers 165 samples (11 biological replicates and five technical)
Data source location	Federal State Institution V. P. Serbsky Federal Medical Research Center of Psychiatry and Narcology National Scientific Research Center on Addictions of the Ministry of Healthcare of the Russian Federation, 119002 Moscow, Russia
Data accessibility	The mass spectrometry proteomics data have been deposited to the ProteomeXchange Consortium via the PRIDE partner repository with the dataset identifier PXD026776. Direct link: https://www.ebi.ac.uk/pride/archive/projects/PXD026776 RAW data available on FTP server: http://ftp.pride.ebi.ac.uk/pride/data/archive/2021/10/PXD026776/

## Value of the Data

• These data are acquired from *in vitro* interaction model of rat glioma and astrocytes cells. This model is helpful for valuable for researchers interested in cancer proteomics.
• Dataset covers eleven biological and five technical replicates in control (rat astrocytes and glioma C-6 without co-cultivating and) and in co-cultivated cells.
• Dataset show the possible mechanisms illustrating how glioma cells can interact with astrocytes cells.

## 1 Data Description

For our co-cultivated *in vitro* model, we used astrocytes and C6 glioma cells. Astrocytes cell lines isolated from rat brain tissue. We analyzed astrocytes in two conditions: before and after co-cultivation. Proteins were assessed in a label-free bottom-up proteomic experiment using IDA approach (i.e. Information Dependent Acquisition) on AB Sciex TripleTOF 6600 Q-TOF mass-spectrometer coupled with LFQ (label-free quantification) approach by MaxQuant software. Dataset covers 165 samples (11 biological and 5 technical replicates) (Table 1).

**Table 1:** Sample description.

Sample name	Description	Biological replicate	Technical replicate
20190704A0001_IDA_1	rat astrocytes	1	1
20190704A0001_IDA_2	rat astrocytes	1	2
20190704A0001_IDA_3	rat astrocytes	1	3
20190704A0001_IDA_4	rat astrocytes	1	4
20190704A0001_IDA_5	rat astrocytes	1	5
20190704A0002_IDA_1	rat astrocytes	2	1
20190704A0002_IDA_2	rat astrocytes	2	2
20190704A0002_IDA_3	rat astrocytes	2	3
20190704A0002_IDA_4	rat astrocytes	2	4
20190704A0002_IDA_5	rat astrocytes	2	5
20190704A0003_IDA_1	rat astrocytes	3	1
20190704A0003_IDA_2	rat astrocytes	3	2
20190704A0003_IDA_3	rat astrocytes	3	3
20190704A0003_IDA_4	rat astrocytes	3	4
20190704A0003_IDA_5	rat astrocytes	3	5
20190704A0004_IDA_1	rat astrocytes	4	1
20190704A0004_IDA_2	rat astrocytes	4	2
20190704A0004_IDA_3	rat astrocytes	4	3
20190704A0004_IDA_4	rat astrocytes	4	4
20190704A0004_IDA_5	rat astrocytes	4	5
20190704A0005_IDA_1	rat astrocytes	5	1
20190704A0005_IDA_2	rat astrocytes	5	2
20190704A0005_IDA_3	rat astrocytes	5	3
20190704A0005_IDA_4	rat astrocytes	5	4
20190704A0005_IDA_5	rat astrocytes	5	5
20190704A0006_IDA_1	rat astrocytes	6	1
20190704A0006_IDA_2	rat astrocytes	6	2
20190704A0006_IDA_3	rat astrocytes	6	3
20190704A0006_IDA_4	rat astrocytes	6	4
20190704A0006_IDA_5	rat astrocytes	6	5
20190704A0007_IDA_1	rat astrocytes	7	1
20190704A0007_IDA_2	rat astrocytes	7	2
20190704A0007_IDA_3	rat astrocytes	7	3
20190704A0007_IDA_4	rat astrocytes	7	4
20190704A0007_IDA_5	rat astrocytes	7	5
20190704A0008_IDA_1	rat astrocytes	8	1
20190704A0008_IDA_2	rat astrocytes	8	2
20190704A0008_IDA_3	rat astrocytes	8	3
20190704A0008_IDA_4	rat astrocytes	8	4
20190704A0008_IDA_5	rat astrocytes	8	5
20190704A0009_IDA_1	rat astrocytes	9	1
20190704A0009_IDA_2	rat astrocytes	9	2
20190704A0009_IDA_3	rat astrocytes	9	3
20190704A0009_IDA_4	rat astrocytes	9	4
20190704A0009_IDA_5	rat astrocytes	9	5
20190704A0010_IDA_1	rat astrocytes	10	1
20190704A0010_IDA_2	rat astrocytes	10	2
20190704A0010_IDA_3	rat astrocytes	10	3
20190704A0010_IDA_4	rat astrocytes	10	4
20190704A0010_IDA_5	rat astrocytes	10	5
20190704A0011_IDA_1	rat astrocytes	11	1
20190704A0011_IDA_2	rat astrocytes	11	2
20190704A0011_IDA_3	rat astrocytes	11	3
20190704A0011_IDA_4	rat astrocytes	11	4
20190704A0011_IDA_5	rat astrocytes	11	5
20190627G0001_IDA_1	rat glioma C6	1	1
20190627G0001_IDA_2	rat glioma C6	1	2
20190627G0001_IDA_3	rat glioma C6	1	3
20190627G0001_IDA_4	rat glioma C6	1	4
20190627G0001_IDA_5	rat glioma C6	1	5
20190627G0002_IDA_1	rat glioma C6	2	1
20190627G0002_IDA_2	rat glioma C6	2	2
20190627G0002_IDA_3	rat glioma C6	2	3
20190627G0002_IDA_4	rat glioma C6	2	4
20190627G0002_IDA_5	rat glioma C6	2	5
20190627G0003_IDA_1	rat glioma C6	3	1
20190627G0003_IDA_2	rat glioma C6	3	2
20190627G0003_IDA_3	rat glioma C6	3	3
20190627G0003_IDA_4	rat glioma C6	3	4
20190627G0003_IDA_5	rat glioma C6	3	5
20190627G0004_IDA_1	rat glioma C6	4	1
20190627G0004_IDA_2	rat glioma C6	4	2
20190627G0004_IDA_3	rat glioma C6	4	3
20190627G0004_IDA_4	rat glioma C6	4	4
20190627G0004_IDA_5	rat glioma C6	4	5
20190628G0005_IDA_1	rat glioma C6	5	1
20190628G0005_IDA_2	rat glioma C6	5	2
20190628G0005_IDA_3	rat glioma C6	5	3
20190628G0005_IDA_4	rat glioma C6	5	4
20190628G0005_IDA_5	rat glioma C6	5	5
20190628G0006_IDA_1	rat glioma C6	6	1
20190628G0006_IDA_2	rat glioma C6	6	2
20190628G0006_IDA_3	rat glioma C6	6	3
20190628G0006_IDA_4	rat glioma C6	6	4
20190628G0006_IDA_5	rat glioma C6	6	5
20190628G0007_IDA_1	rat glioma C6	7	1
20190628G0007_IDA_2	rat glioma C6	7	2
20190628G0007_IDA_3	rat glioma C6	7	3
20190628G0007_IDA_4	rat glioma C6	7	4
20190628G0007_IDA_5	rat glioma C6	7	5
20190628G0008_IDA_1	rat glioma C6	8	1
20190628G0008_IDA_2	rat glioma C6	8	2
20190628G0008_IDA_3	rat glioma C6	8	3
20190628G0008_IDA_4	rat glioma C6	8	4
20190628G0008_IDA_5	rat glioma C6	8	5
20190628G0009_IDA_1	rat glioma C6	9	1
20190628G0009_IDA_2	rat glioma C6	9	2
20190628G0009_IDA_3	rat glioma C6	9	3
20190628G0009_IDA_4	rat glioma C6	9	4
20190628G0009_IDA_5	rat glioma C6	9	5
20190628G0010_IDA_1	rat glioma C6	10	1
20190628G0010_IDA_2	rat glioma C6	10	2
20190628G0010_IDA_3	rat glioma C6	10	3
20190628G0010_IDA_4	rat glioma C6	10	4
20190628G0010_IDA_5	rat glioma C6	10	5
20190628G0011_IDA_1	rat glioma C6	11	1
20190628G0011_IDA_2	rat glioma C6	11	2
20190628G0011_IDA_3	rat glioma C6	11	3
20190628G0011_IDA_4	rat glioma C6	11	4
20190628G0011_IDA_5	rat glioma C6	11	5
20190708RA0001_IDA_1	astrocytes co-cultivation with glioma C6	1	1
20190708RA0001_IDA_2	astrocytes co-cultivation with glioma C6	1	2
20190708RA0001_IDA_3	astrocytes co-cultivation with glioma C6	1	3
20190708RA0001_IDA_4	astrocytes co-cultivation with glioma C6	1	4
20190708RA0001_IDA_5	astrocytes co-cultivation with glioma C6	1	5
20190708RA0002_IDA_1	astrocytes co-cultivation with glioma C6	2	1
20190708RA0002_IDA_2	astrocytes co-cultivation with glioma C6	2	2
20190708RA0002_IDA_3	astrocytes co-cultivation with glioma C6	2	3
20190708RA0002_IDA_4	astrocytes co-cultivation with glioma C6	2	4
20190708RA0002_IDA_5	astrocytes co-cultivation with glioma C6	2	5
20190708RA0003_IDA_1	astrocytes co-cultivation with glioma C6	3	1
20190708RA0003_IDA_2	astrocytes co-cultivation with glioma C6	3	2
20190708RA0003_IDA_3	astrocytes co-cultivation with glioma C6	3	3
20190708RA0003_IDA_4	astrocytes co-cultivation with glioma C6	3	4
20190708RA0003_IDA_5	astrocytes co-cultivation with glioma C6	3	5
20190708RA0004_IDA_1	astrocytes co-cultivation with glioma C6	4	1
20190708RA0004_IDA_2	astrocytes co-cultivation with glioma C6	4	2
20190708RA0004_IDA_3	astrocytes co-cultivation with glioma C6	4	3
20190708RA0004_IDA_4	astrocytes co-cultivation with glioma C6	4	4
20190708RA0004_IDA_5	astrocytes co-cultivation with glioma C6	4	5
20190715RA0005_IDA_1	astrocytes co-cultivation with glioma C6	5	1
20190715RA0005_IDA_2	astrocytes co-cultivation with glioma C6	5	2
20190715RA0005_IDA_3	astrocytes co-cultivation with glioma C6	5	3
20190715RA0005_IDA_4	astrocytes co-cultivation with glioma C6	5	4
20190715RA0005_IDA_5	astrocytes co-cultivation with glioma C6	5	5
20190715RA0006_IDA_1	astrocytes co-cultivation with glioma C6	6	1
20190715RA0006_IDA_2	astrocytes co-cultivation with glioma C6	6	2
20190715RA0006_IDA_3	astrocytes co-cultivation with glioma C6	6	3
20190715RA0006_IDA_4	astrocytes co-cultivation with glioma C6	6	4
20190715RA0006_IDA_5	astrocytes co-cultivation with glioma C6	6	5
20190715RA0007_IDA_1	astrocytes co-cultivation with glioma C6	7	1
20190715RA0007_IDA_2	astrocytes co-cultivation with glioma C6	7	2
20190715RA0007_IDA_3	astrocytes co-cultivation with glioma C6	7	3
20190715RA0007_IDA_4	astrocytes co-cultivation with glioma C6	7	4
20190715RA0007_IDA_5	astrocytes co-cultivation with glioma C6	7	5
20190715RA0008_IDA_1	astrocytes co-cultivation with glioma C6	8	1
20190715RA0008_IDA_2	astrocytes co-cultivation with glioma C6	8	2
20190715RA0008_IDA_3	astrocytes co-cultivation with glioma C6	8	3
20190715RA0008_IDA_4	astrocytes co-cultivation with glioma C6	8	4
20190715RA0008_IDA_5	astrocytes co-cultivation with glioma C6	8	5
20190716RA0009_IDA_1	astrocytes co-cultivation with glioma C6	9	1
20190716RA0009_IDA_2	astrocytes co-cultivation with glioma C6	9	2
20190716RA0009_IDA_3	astrocytes co-cultivation with glioma C6	9	3
20190716RA0009_IDA_4	astrocytes co-cultivation with glioma C6	9	4
20190716RA0009_IDA_5	astrocytes co-cultivation with glioma C6	9	5
20190716RA0010_IDA_1	astrocytes co-cultivation with glioma C6	10	1
20190716RA0010_IDA_2	astrocytes co-cultivation with glioma C6	10	2
20190716RA0010_IDA_3	astrocytes co-cultivation with glioma C6	10	3
20190716RA0010_IDA_4	astrocytes co-cultivation with glioma C6	10	4
20190716RA0010_IDA_5	astrocytes co-cultivation with glioma C6	10	5
20190716RA0011_IDA_1	astrocytes co-cultivation with glioma C6	11	1
20190716RA0011_IDA_2	astrocytes co-cultivation with glioma C6	11	2
20190716RA0011_IDA_3	astrocytes co-cultivation with glioma C6	11	3
20190716RA0011_IDA_4	astrocytes co-cultivation with glioma C6	11	4
20190716RA0011_IDA_5	astrocytes co-cultivation with glioma C6	11	5

## 2 Experimental Design, Materials and Methods

### 2.1 Cultivation

C6 glioma and astrocytes cell lines isolated from rat brain tissue were grown in 25 cm^2^ Corning culture flasks. As the growth medium, we used RPMI-1640 (Thermo Fisher Scientific) with the addition of fetal bovine serum (10% of the total volume of the medium), sodium pyruvate (ml/100 ml of medium), L-glutamine (ml/100 ml of medium) and a solution of an antibiotic (ml/100 ml of medium) containing 10,000 IU/ml of penicillin, 10,000 µg/ml of streptomycin and 25 µg/ml of amphotericin B. The growth medium was changed every 2,3 days. Upon reaching the monolayer, cells were dissociated from the culture vials using a 0.25% trypsin solution followed by 3-fold washing with Dubelco's phosphate-saline buffer. Cell counting was performed in a Goryaev chamber and on a TC20TM Automated Cell Counter (BIO-RAD) automatic cell counter.

To obtain samples of co-cultured astrocytes, joint cultivation of astrocytes and C6 glioma cells was performed. For this, astrocytes were seeded in 6-well Corning Costar plates in the amount of 300 thousand cells per well. C6 glioma cells were sown in special inserts at a concentration of 50 thousand cells per well. Co-cultivation was carried out for 10 days. The growth medium was updated every 2,3 days. On the 10th day, the inserts were removed, astrocytes were dissociated from the culture plates, and samples were prepared as described above.

### 2.2 Sample preparation

All reagents used to perform sample preparation were manufactured by Sigma-Aldrich and have a grade of purity of OCP, unless otherwise specified.

15 µl of 10% sodium deoxycholate was added to the resulting cell pellet, after which the pellet was placed on a vortex. Then, 1 µl of nuclease mix (GE Healthcare) was added to the pellet, after which the solution was placed on a vortex and intubated for 1 hour at 4°C.

Next, a solution containing 100 µl of 100 mM Tris-HCl, 0.05% sodium deoxycholate, 2.5 mM EDTA and 8 M urea (pH 8) was added to the sample. The solution was incubated for 30 minutes at room temperature (RT). After incubation, the sample was centrifuged at 14,000 rpm for 10 min at 20°C (Eppendorf, Centrifuge 5810). After all, the supernatant was selected. The actual abundance of proteins was measured with the Bradford (ThermoFisher, USA) method for protein quantitation. Next, tris (2-carboxyethyl) phosphine (TCEP) was added to the supernatant to a final concentration of 5 mM. The resulting solution was incubated for 1 h at 37°C. Then, iodoacetamide (IAA) was added to the resulting solution to a final concentration30 mM. The resulting solution was incubated for 30 min in the dark at RT. Finally, TCEP was added to the sample to a final concentration 2.5 mM and the sample was incubated for 20 min at RT. The resulting sample solution was diluted with 6 × volume to a final urea concentration 1.5 M and containing 50 mM Tris-HCl and 0.05% DCNa (pH 8). Trypsin (Promega, MS grade) (1 µg / µl) in a ratio of 1:50 (trypsin: protein) was added to the resulting sample. The resulting solution was incubated for 16 h at 37°C.

After tryptic digestion (Promega, USA), a solution of 50% trifluoroacetic acid (Sigma, USA) was added to the sample to pH 2.0. After that samples were centrifuged, and the supernatant was taken. The supernatant was purified by solid phase extraction using Discovery DSC-18 Tube (Supelco) columns according to the manufacturer's protocol. Peptides were eluted from a 1 ml column of buffer containing 50% acetonitrile and 0.1% TFA.

The resulting peptide extract was dried on a vacuum concentrator (Labconco) almost to dryness and resuspended in a buffer containing 3% acetonitrile and 0.1% TFA to a final concentration of native protein of 5 µg/ml.

### 2.3 LC-MS/MS analysis

The LC-MS/MS analysis of tryptic peptides was performed using a microflow HPLC system Eksigent Ekspert (Sciex, USA) with autosampler Eksigent Ekspert 400 (Sciex, USA) coupled to AB Sciex Triple TOF 6600 with microflow AB Sciex Duo Spray ion Source (Sciex, USA). The chromatographic separation of the peptides was performed with Eksigent column, 2.7 um, HALO Fused-Core C18, 50 × 0.5 mm 805-10100. Chromatographic separation was performed with the parameters: (parameters). The ion source settings were as follow: the flow rate of nebulizer gas - 10 L/min; the voltage applied to the capillary 4500 V. The mod of obtaining MS spectra is as follows: accumulation time 50 ms, mass/charge range from 100 to 2500 m/z, criteria of preferred selection of ions for isolation and fragmentation - charge 2–5, number parent ions from one MS spectrum - 25, the minimum intensity is 5000, resolution 30,000. After the first analysis, the ions were excluded from the candidate for fragmentation 5 s. The fragmentation parameters were as follows: the accumulation time of the spectra fragmentation - 20 ms, charge/mass ratio from 100 to 2500 m/z, full cycle time 600 ms, resolution 25,000.

### 2.4 Protein identification and quantification analysis

Identification and label-free quantification analysis were performed with MaxQuant software with default settings against a database of all proteins of *Rattus norvegicus* (Uniprot) of 2020. All proteins in database are reviewed by Swiss-Prot. Human keratins were added to all databases to avoid misinterpretation of contaminating proteins.

### 2.5 Analysis and data processing

The data was processed using the software SCIEX Analyst, SCIEX PeakView, SCIEX ProteinPilot, MaxQuant, RStudio, OmicsBox. All identifications are performed with follow parameters: protein and peptide FDR – 1%, fixed modification – Carbamidomethyl (C), variable modification – oxidation (M), Acetyl (Protein N-term), MS and MSMS mass tolerance – 20 ppm, max number of misscleavages site – 2. Other specific parameters of search in mqpar.xml file.

### 2.6 Results

As a result of the analysis, 1567 proteins for astrocytes (A) were quantified; 1667 for glioma (G); 1684 for co-cultured astrocytes (RA). In total, 2708 proteins were quantified for all three biological groups.

The result was considered reliable if in a pairwise comparison between the three biological groups, the signal intensity for the protein was more than doubled and the p-value adjusted by Benjamini-Hochberg was less than 0.05. Thus, a change in the level of 162 proteins (G / A) was reliably established between glioma and astrocytes; between co-cultured astrocytes and glioma 141 protein (RA/G); between co-cultured astrocytes and astrocytes, a change of 70 proteins (RA / A) was found.

## Ethics Statement

Animals were not used in the experiment. C6 glioma cells and rat astrocytes were purchased from the American Type Culture Collection (ATCC) and were stored and cultured according to the manufacturer's protocols.

## CRediT authorship contribution statement

**Artemiy S. Silantyev:** Writing – original draft, Formal analysis. **Olga N. Bukato:** Writing – review & editing, Formal analysis. **Ivan O. Butenko:** Software. **Anastasia A. Chernysheva:** Methodology. **Olga V. Pobeguts:** Methodology, Resources. **Alexander E. Nosyrev:** Conceptualization. **Olga I. Gurina:** Resources, Funding acquisition, Project administration.

## Declaration of Competing Interest

The authors declare that they have no known competing financial interests or personal relationships that could have appeared to influence the work reported in this paper.

## Appendix Supplementary materials

Image, application 1

Image, application 2
